# A SAFT Method for the Detection of Void Defect inside a Ballastless Track Structure Using Ultrasonic Array Sensors

**DOI:** 10.3390/s19214677

**Published:** 2019-10-28

**Authors:** Wen-Fa Zhu, Xing-Jie Chen, Zai-Wei Li, Xiang-Zhen Meng, Guo-Peng Fan, Wei Shao, Hai-Yan Zhang

**Affiliations:** 1 School of Urban Rail Transportation, Shanghai University of Engineering Science, Shanghai 201620, China; chenxingjie@sues.edu.cn (X.-J.C.); zaiweili@sues.edu.cn (Z.-W.L.); M105117117@sues.edu.cn (X.-Z.M.); M105118102@sues.edu.cn (W.S.); 2 School of Communication and Information Engineering, Shanghai University, Shanghai 200444, China; phdfanry@shu.edu.cn (G.-P.F.); hyzhang@shu.edu.cn (H.-Y.Z.)

**Keywords:** SAFT imaging, ultrasonic array sensors, multilayer structure, ballastless track structure, void defect detection

## Abstract

High-precision ultrasound imaging of void defects is critical for the performance and safety assessment of ballastless track structures. The sound propagation velocity of each layer in the ballastless track structure is quite different. However, the traditional concrete Synthetic Aperture Focusing Technique (SAFT) ultrasound imaging method is based on the assumption that the concrete has a single constant shear wave velocity. Thus, it is not a suitable method for the ultrasonic imaging of multilayer structures. In this paper, a Multilayer SAFT high-precision ultrasound imaging method is proposed. It is based on the ray-tracing technique and uses the Fermat principle to find the refraction point that minimizes the delay of the acoustic wave propagation path at the interface of the discrete layers. Then, the acoustic wave propagation path is segmented by the position of the refraction point, and the propagation delay of the ultrasonic wave is obtained segment by segment. Thus, the propagation delay of the ultrasonic wave is obtained one by one, so that the propagation delay of the ultrasonic wave in the multilayer structure can be accurately obtained. Finally, the focused image is obtained according to the SAFT imaging algorithm. The finite element simulation and experimental results show that the Multilayer SAFT imaging method can accurately track the propagation path of the ultrasonic wave in ballastless track structures, as well as accurately calculate the propagation delay of the ultrasonic wave and the lengths of void defects. The high accuracy of the Multilayer SAFT imaging represents a significant improvement compared to traditional SAFT imaging.

## 1. Introduction

By the end of 2018, the total length of the Chinese high-speed railway reached 29,000 km [1]. The ballastless track structure is the main track structure of China’s high-speed rail lines, accounting for more than 50% of the operating lines [2]. The high-speed rail ballastless track structure is a typical multilayer structure in which the track slab (concrete material) and bedplate (concrete material) are bonded together by cement, asphalt, and mortar [3]. The high-speed rail ballastless track structure is an important bearing component. Under the influence of dynamic load, temperature, and other environmental factors, the phenomenon of void defects will frequently occur [4]. Taking the Shanghai–Hangzhou high-speed railway in the Shanghai Railway Administration as an example, a total of 3912 defects were found in 2018. Void defects have become a major source of danger affecting the safety of high-speed railways. Therefore, the accurate detection of void defects in ballastless track structures has become one of the core problems that urgently need to be solved for the maintenance and repair of high-speed railway line structures in China.

Ultrasonic testing is one of the effective methods for detecting internal defects in concrete structures. The interpretation of ultrasonic testing data is crucial. Traditional A-scan analysis methods, such as time–frequency analysis [5], wavelet transform analysis [6], and time-of-flight analysis [7], can be used for signal feature analysis and defect detection of concrete structures. However, these methods can only determine whether there are defects in the concrete, but cannot detect the position and size of the defects [8,9]. The high-precision positioning, quantitative evaluation, and visual representation of void defects are crucial for the service performance and safety assessment of ballastless track structures. This context is also a hotspot for ultrasonic nondestructive testing [10]. The Synthetic Aperture Focusing Technique (SAFT) is a mature industrial ultrasonic imaging method that has been widely used in the detection of internal defects of concrete structures such as bridge decks [11], concrete columns [12], pavement [13], tunnels [14], etc. As early as the 1950s, SAFT began to be successfully applied in the radar field [15]. In the 1970s, Frederick et al. of the University of Michigan successfully applied SAFT to the field of ultrasonic nondestructive testing [16]. Since then, SAFT has been developed rapidly for the field of ultrasonic testing [17]. However, the existing concrete SAFT ultrasonic imaging method is based on the assumption that the concrete is a uniform medium with a single constant shear wave velocity, and the defects are ultrasonically imaged [18]. However, high-speed railway ballastless track structures are multilayer structures, and the acoustic impedance difference between layers is not negligible [19,20]. In this case, the assumption of a single constant shear wave velocity is bound to produce detection errors [21,22].

The SAFT method can relatively easily achieve ultrasound imaging of a single medium, however, this method is much more complicated when it comes to calculating multilayer structures [23]. The SAFT imaging methods for multilayer structures mainly include the virtual sound source method [24], root-mean-square velocity method [25], phased shift migration method [26], and ray tracing technique [27]. The virtual sound source method is only applicable to two-layer plate structures, and it must take into consideration that the sound field assumed by the focus analog sound source may deviate from the actual probe acoustic emission. Therefore, imaging improvement using this method is limited. The root-mean-square velocity method has a higher imaging accuracy for multilayer structures with similar sound velocities. However, when the sound velocity of multilayer structures differs greatly, the imaging resolution of this method will decrease when it deviates from the second-order Taylor series approximation. The phased shift migration method greatly improves the imaging efficiency of multilayer structures as compared to the frequency domain SAFT method, but this method is generally based on the development of the planar probe sound propagation characteristics, and it is difficult to apply to a focusing probe [28]. 

The ray-tracing technique can effectively find the propagation path of sound waves in a multilayer structure, so the propagation delay of sound waves in the structures can be accurately determined. Therefore, this paper proposes a multilayer SAFT imaging method based on the ray-tracing technique to achieve high-precision ultrasound imaging of void defects in a ballastless track structure. Firstly, the multilayer SAFT imaging algorithm based on the ray-tracing technique is studied. The Fermat principle is used to find the refraction point, which makes the acoustic wave propagation path delay the shortest at the discrete layer-to-layer interface, and the acoustic wave propagation path is located at the refraction point position. Through segmentation, the propagation delay of the ultrasonic wave is obtained bit by bit, so that the propagation delay of the ultrasonic wave in the multilayer structure can be accurately obtained. Finally, the focused image is obtained according to the Delay-And-Sum (DAS) algorithm. A two-dimensional finite element model of ultrasonic wave propagation in ballastless track structures is then established. By obtaining the full wavefield data in the finite element model, the imaging quality and precision of the traditional SAFT imaging method and multilayer SAFT imaging method for void defects in a ballastless track structure are analyzed. Finally, the Russian MIRA-A1040 concrete ultrasonic tomography scanner is used to acquired ultrasonic echo signals in a ballastless track structure, and the effectiveness of multilayer SAFT imaging is verified by experiments.

## 2. Ballastless Track Structure Multilayer Synthetic Aperture Focusing Technique Method

### 2.1. SAFT Imaging Method

**Figure 1:**
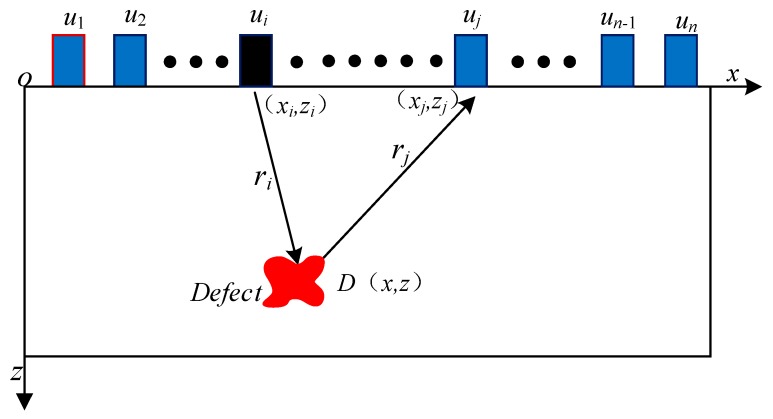
Synthetic Aperture Focusing Technique (SAFT) for a single-layer medium.

The principle of the SAFT imaging method is shown in Figure 1. In a group of ultrasonic transducer arrays, ultrasonic waves are sequentially emitted by one probe. The other probes receive ultrasonic waves. The received ultrasonic echo signals are processed by DAS. Lastly, a reconstructed B-scan image is produced.

When information such as the position and size of the defects in the object to be measured are unknown, SAFT imaging can be used to calculate the value of all the pixels by traversing the imaging region.

As shown in Figure 1, it is assumed that the sensor position is *u_n_* (*n* = 1, 2, 3...*n*), while the exciting sensor position coordinates are (*x_i_*, *z_i_*). The receiving sensor position coordinates are (*x_j_*, *z_j_*). The position coordinate of any pixel *D* is (*x*, *z*). The time delay *t_ij_* (*x*, *z*) experienced by the ultrasonic wave is transmitted from the position of the exciting sensor *u_i_* and then reaches the position of the receiving sensor *u_j_* via point *D*, as shown in Equation (1):

(1) $$t_{ij}(x,z)=\frac{r_{i}+r_{j}}{v}$$

where the *r_i_* is given by:

$$r_{i}=\sqrt{{\left(x-x_{i}\right)}^{2}+{\left(z-z_{i}\right)}^{2}},\;r_{j}=\sqrt{{\left(x-x_{j}\right)}^{2}+{\left(z-z_{j}\right)}^{2}},$$

and *v* is the speed at which sound waves travel in the object being measured, *i*, *j* = 1,2,3…*n*.

When the exciting and receiving transducer positions are *u_i_* and *u_j_*, respectively, the value of any pixel *D* (*x*, *z*) can be expressed as Equation (2):

(2) $$I_{ij}(x,z)=S_{ij}\left(t_{ij}(x,z)\right)$$

where *I_ij_* (*x*, *z*) represents the value of pixel *D* (*x*, *z*) when the exciting and receiving transducer positions are *u_i_* and *u_j_*. *S_ij_* represents the amplitude envelope of the received signal when the exciting and receiving transducer positions are *u_i_* and *u_j_*.

According to Equation (3), the value of any pixel can be obtained in the imaging area grid:

(3) $$I^{\prime }\left(x,z\right)=\sum_{i=1}^{n}\sum_{j=1}^{n}I_{ij}(x,z).$$

### 2.2. Multilayer SAFT Imaging Method Based on Ray Tracing Technique

The SAFT imaging method makes it easier to achieve the ultrasound imaging of a single medium object, while for multilayer structures like ballastless track structures, ultrasound imaging calculations are much more complicated. The ultrasonic phenomenon occurs when the ultrasonic wave penetrates the interface between two adjacent layers of multilayer structures, and the sound propagation path at this time is composed of the refracting line segments in each layer. According to the conventional SAFT calculation model, the ultrasonic propagation delay is calculated using Equation (1). For multilayer structures (especially when the wave velocity of each layer medium is large), the calculation of the propagation delay using Equation (1) will produce a large error.

**Figure 2:**
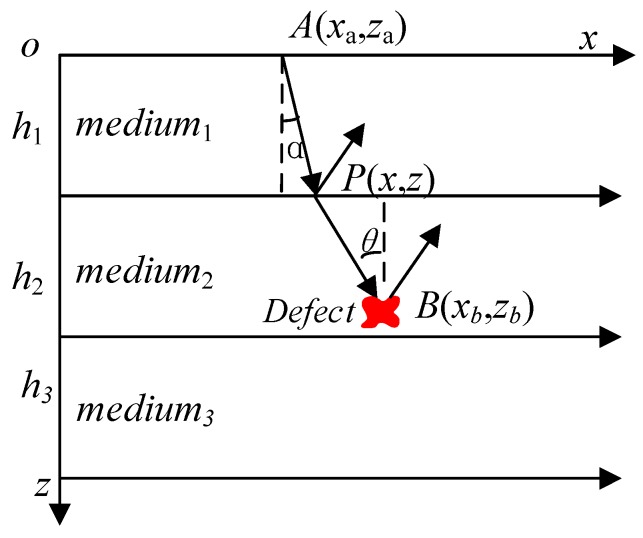
Acoustic wave propagation path.

The difficulty of SAFT imaging in multilayer structures lies in accurately obtaining the propagation delay of acoustic waves, that is, accurately obtaining the propagation path. The ray-tracing technique is one of the effective methods to solve this problem. As shown in Figure 2, *α* is the incident angle of the acoustic wave and *θ* is the angle of refraction of the acoustic wave. The time at which the acoustic wave is refracted from point *A* through point *P* and propagates to point *B* can be expressed by Equation (4).

(4) $$t=\frac{\sqrt{{\left(x-x_{a}\right)}^{2}+{\left(z-z_{a}\right)}^{2}}}{v_{1}}+\frac{\sqrt{{\left(x-x_{b}\right)}^{2}+{\left(z-z_{b}\right)}^{2}}}{v_{2}}$$

where *t* is the total delay of the acoustic wave propagating from *A* to *B*, *v*_1_ is the speed of sound in the first layer of the medium, and *v*_2_ is the speed of sound in the second layer of the medium.

The ray-tracing technique is a method to calculate the acoustic wave propagation time according to Snell’s law or Fermat’s shortest time principle. The actual path of soundwave from point a to point B conforms to Snell’s Law (Equation (5)). At the same time, according to Fermat’s principle, the actual acoustic wave propagation time from point A to point B is the shortest.

(5) $$\frac{sin\alpha }{v_{1}}=\frac{sin\theta }{v_{2}}.$$

**Figure 3:**
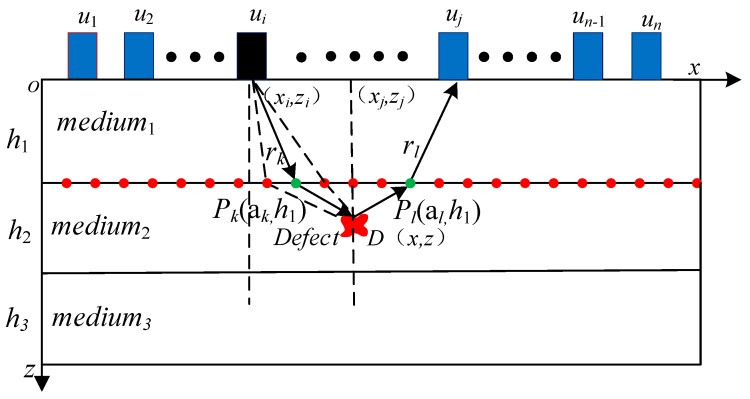
Multilayer SAFT imaging.

As shown in Figure 3, in order to calculate the time when the acoustic wave reaches a certain point D of the second layer, firstly, the traversal method is used to find the refraction point that makes the acoustic wave propagation path delay the shortest at the interface of the discrete layer. Then the acoustic wave propagation path is segmented by the position of the refraction point, and the ultrasonic propagation is obtained one by one. When the propagation delay of the ultrasonic wave in the multilayer structure is accurately obtained, a focused image can finally be obtained according to the DAS algorithm. The specific algorithm is as follows:

Step 1: Calculate the acoustic wave from the probe *u_i_* through point *P_k_* to the pixel point of the refractive time *t_ik_* of *D* using Equation (6).

Step 2: Obtain the time delay *t_i_* of the refractive propagation path from Equation (7).

Step 3: Calculate the total time delay *t_ij_*(*x*, *z*) for the acoustic wave that propagates from sensor *u_i_* through pixel point *D* in the second layer medium to sensor *u_j_* according to Equation (8).

(6) $$t_{ik}(x,z)=\frac{\sqrt{{\left(x_{i}-a_{k}\right)}^{2}+{\left(z_{i}-h_{1}\right)}^{2}}}{v_{1}}+\frac{\sqrt{{\left(x-a_{k}\right)}^{2}+{\left(z-h_{1}\right)}^{2}}}{v_{2}}$$

(7) $$t_{i}(x,z)=min\left[t_{ik}(x,z)\right]$$

(8) $$t_{ij}(x,z)=t_{i}(x,z)+t_{j}(x,z)$$

Combining the ray-tracing technique with the SAFT imaging method can solve the problem of the traditional SAFT imaging method, which is not applicable to multilayer structures due to the change of the refractive propagation path when the acoustic wave propagates through the layer interfaces. With the proposed method, however, high-resolution imaging of a multilayer structure can be realized. The value in the second layer medium can be calculated by using Equation (9):

(9) $$I^{\prime \prime }(x,z)=\overset{n}{\underset{i=1}{\sum }}\overset{n}{\underset{j=1}{\sum }}S_{ij}\left(t_{ij}(x,z)\right)$$

where *z* > *h*_1_, *I*″ (*x*, *z*) represents the value of pixel point (*x*, *z*) in the second layer medium and *S_ij_* represents the amplitude envelope curve of the received signal when the exciting and receiving sensor positions are *u_i_* and *u_j_*, respectively.

## 3. Finite Element Simulation

### 3.1. Finite Element Model

**Figure 4:**
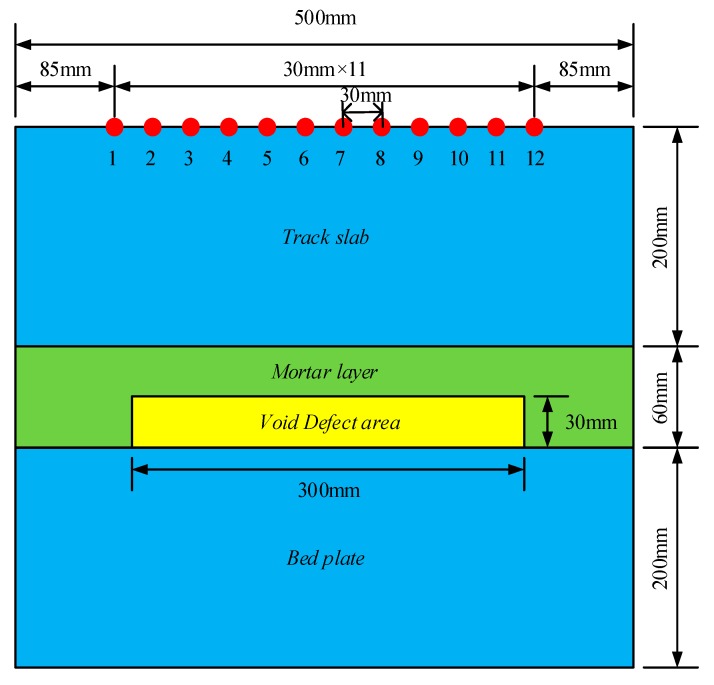
Two-dimensional finite element model of ballastless track structures.

The commercial sound field finite element software PZFlex was used to establish a two-dimensional finite element simulation model of ballastless track structures. As shown in Figure 4, the finite element model is divided into a first layer for the track plate from the top to the bottom, a second layer for the mortar layer, and a third layer for the bedplate. The material parameters of each layer are shown in Table 1. Twelve transducer positions are arranged on the surface of the track plate, and the distance between the transducers is 30 mm. A void area of 300 mm in length and 30 mm in width was placed in the mortar layer.

In order to avoid the boundary reflection wave affecting the imaging result, the four boundaries of the model were set as the absorption boundaries. The specific finite element parameter settings are shown in Table 2. The excitation signal used in this paper was a three-cycle sine wave modulated by the Hanning window with 50 kHz central frequency. 

**Figure 5:**
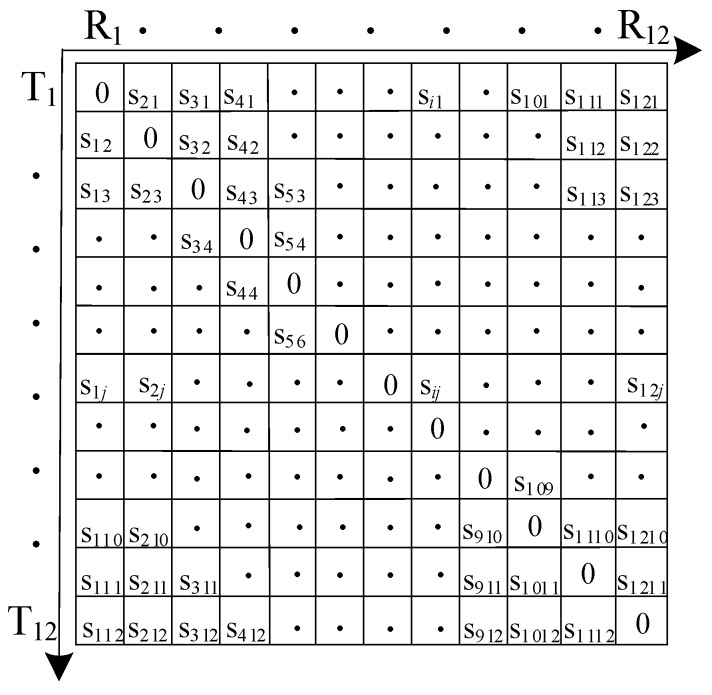
Full wavefield data.

As shown in Figure 5, the data are collected by one transmission and multiple collections, that is, one of the 12 array transducers is used as an exciting source to excite the transverse wave signal in the track slab, and the remaining 11 transducers receive the signals and collect a total of 132 sets of data.

### 3.2. Analysis of Finite Element Simulation Results

**Figure 6:**
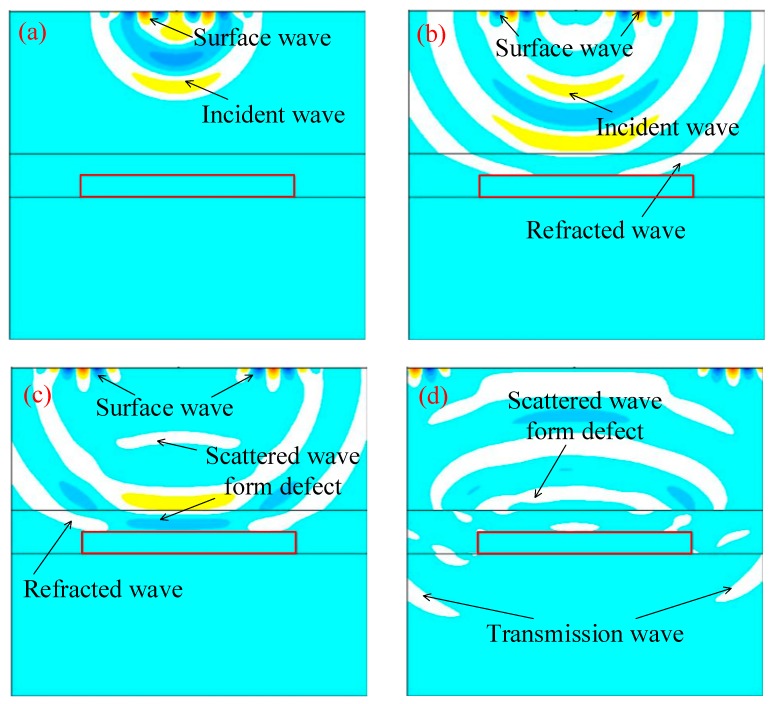
Snapshots of wavefield (**a**) 49.8 μs, (**b**) 69.7 μs, (**c**) 89.6 μs, and (**d**) 129.4 μs (the red rectangular area is a void defect).

Figure 6 shows that the transient sound field distribution at each moment in the finite element simulation process. Figure 6 is a transient sound field distribution diagram, showing the measurements at 49.8, 69.7, 89.6, and 129.4 μs at the time when the sixth sensor is employed as the excitation source.

**Figure 7:**
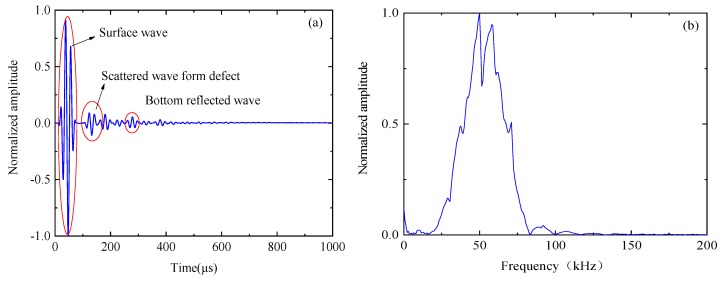
Single received signal: (**a**) time-domain and (**b**) spectrum.

**Figure 8:**
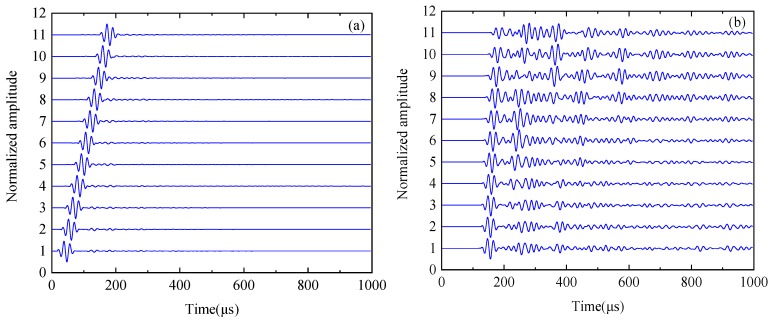
Time-domain: (**a**) the original signal (including surface waves) and (**b**) the signal after removing the surface wave.

Figure 7 shows the time and frequency domains of the first sensor receiving the signal as the excitation source. The transient sound field distribution of Figure 7 shows that the received signal contains a larger energy surface wave. The presence of this surface wave signal will seriously affect the image quality. In the case of finite element simulation, the signal obtained by the finite element model under healthy conditions is used as the reference signal, and the defect scattering signal is obtained by subtracting the reference signal from the finite element model simulation signal containing the defect, thereby eliminating the influence of the surface wave. Figure 8 is a time-domain of a set of signals. Figure 8a is the original signal (including surface waves). Figure 8b is the signal after removing the surface wave.

**Figure 9:**
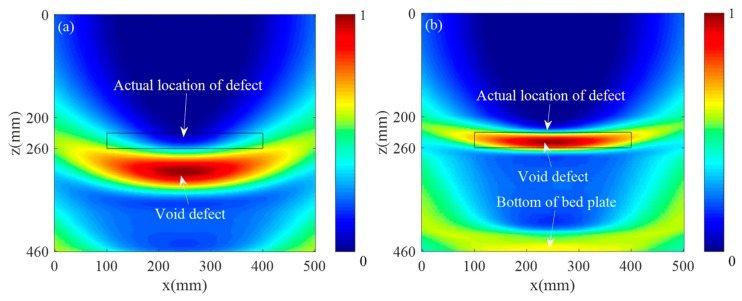
Ultrasound imaging results: (**a**) SAFT imaging and (**b**) multilayer SAFT imaging.

**Figure 10:**
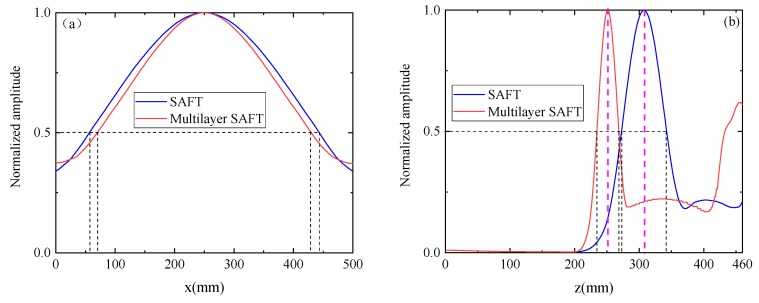
Sectional view of the imaging results: (**a**) *x*-direction and (**b**) *z*-direction.

The collected 132 sets of defect scattering signals were ultrasonically imaged using the traditional SAFT imaging algorithm and the multilayer SAFT imaging algorithm, separately. Figure 9a shows the imaging results obtained by the conventional SAFT algorithm. It can be seen that when imaging is performed using only a single wave velocity, the imaging position of the defect differs greatly from the actual position. This is because the wave velocity of the track slab and the bonding layer are greatly different, and the propagation delay of the ultrasonic wave is calculated by using a single wave velocity. Figure 9b shows the imaging results obtained by the Multilayer SAFT algorithm. It can be seen that the position of the defect is in good agreement with the actual position due to the consideration of the propagation wave velocity of different ultrasonic waves in different media. At the same time, there are still some areas where the value is higher on the lower side of the defect area. This phenomenon is caused by the underlying reflected wave generated when the sound wave propagates to the bottom layer. Figure 10 shows a cross-sectional view of the imaging results of the two methods in the *x* and *z*-directions. As can be seen from Figure 10a, the defect length range of the imaging result by the SAFT algorithm is (57.3 mm, 443 mm), the error is 85.7 mm, the length center position is *x* = 250 mm, and the error is 0 mm. The defect length range of the imaging results using the Multilayer SAFT algorithm is (70.7 mm, 428.2 mm) with an error of 57.5 mm, the center position of the length is *x* = 250 mm, and the error is 0 mm. From Figure 10b, the height range of the imaging result by the SAFT algorithm is (270.8 mm, 340.3 mm), the error is 39.5 mm, the *z*-direction center position is *z* = 307.7 mm, and the error is 62.7 mm. The height range of the imaging results using the Multilayer SAFT algorithm is (232.6 mm, 265.3 mm) with an error of 2.7 mm, the center position in the *z*-direction is *z* = 251.6 mm, and the error is 6.6 mm. It can be seen from the above results that the Multilayer SAFT algorithm considering the propagating wave velocity in different media has better imaging accuracy than the SAFT imaging algorithm using single wave velocity. The length characterization accuracy is improved by 32.9% and the height characterization accuracy is improved by 93.2%. The center characterization accuracy of the defect in the z-direction is improved by 93.2%.

## 4. Experimental Verification

### 4.1. Experimental System

**Figure 11:**
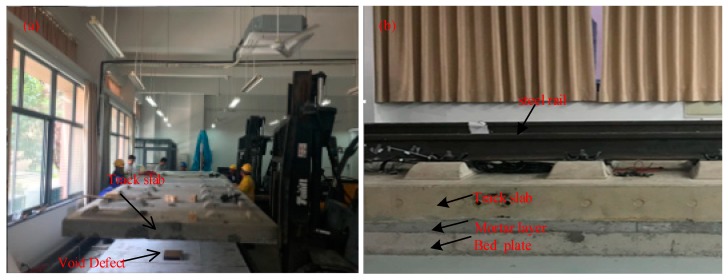
Model of ballastless track structure: (**a**) construction process and (**b**) structure side view.

A 1:1 model of the ballastless track structure, as shown in Figure 11, was established in the laboratory to verify the accuracy of the algorithm studied in this paper. The material ratio and materials of each layer in the 1:1 model of the ballastless track structure were strictly in accordance with the “Code for Design of Durability of Railway Concrete Structures” (TB 10005-2010), and the construction process was conducted in accordance with “CRTSII type slab ballastless track concrete track slab” (TB/T 3399-2015) production. In the production process of the 1:1 model, a void defect with a length of 300 mm, a width of 300 mm, and a height of 15 mm, was pre-buried at the interface between the second layer and the third layer.

**Figure 12:**
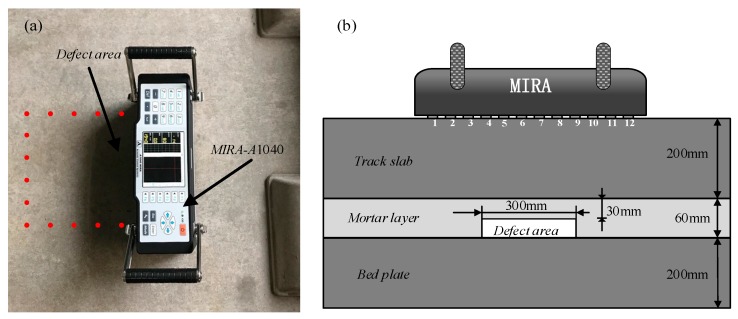
Experimental system and signal acquisition mode: (**a**) Experimental system; (**b**) Schematic of the signal acquisition mode.

In the experiment, the ultrasonic echo signal was collected by Russian MIRA-A1040 concrete ultrasonic tomography, as shown in Figure 12a, and the acquired signal was exported and subjected to signal processing and ultrasonic imaging in MATLAB software. The MIRA-A1040 consists of 48 drypoint contact (DPC) transmitting and receiving transducers mounted in a matrix antenna. As shown in Figure 12b, the MIRA-A1040 array has 12 channels spaced 30 mm apart. Each channel has four transducers with a lateral spacing of 25 mm. In the data acquisition, when 12 channels are sequentially used as the transmitter of the transverse wave in the i-th channel, the 12-i channels after the channel are used as receivers to collect data, so that 66 sets of data can be collected. The sampling frequency used in the experiment was 1 MHz and the probe center frequency was 50 kHz.

### 4.2. Method of Suppressing Surface Wave

**Figure 13:**
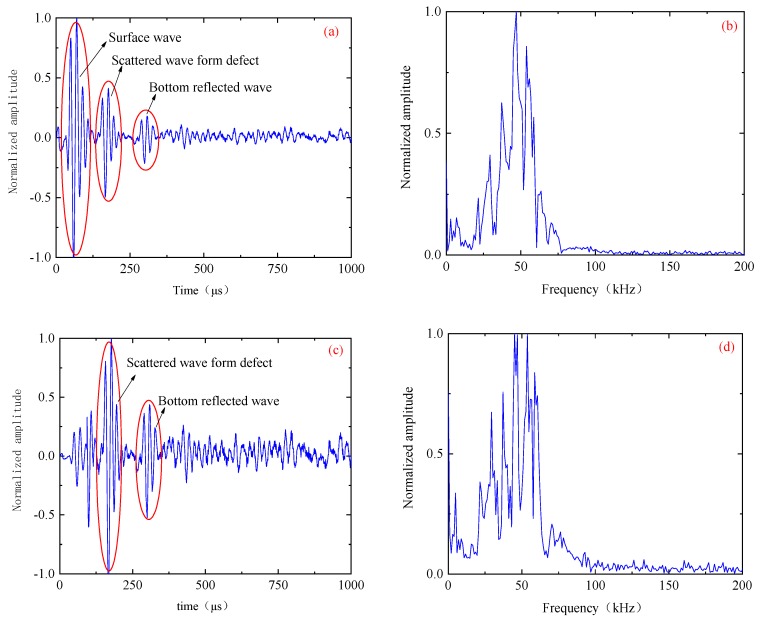
The ultrasonic signal: (**a**) original experimental signal containing surface waves, defective scattered waves, and bottom reflected waves, (**b**) original signal spectrum, (**c**) signal after surface wave removal, and (**d**) spectrum for removing surface wave signals.

Figure 13a depicts a set of original experimental signals containing surface waves, defective scattered waves, and bottom reflected waves. It can be seen from the time domain characteristics of the signals that the surface wave signal and the defect scattering signal are separated from each other in the time-domain so that the surface wave can be directly suppressed by windowing the time domain.

Using Equation (10), the surface wave velocity *V_r_* of the track slab can be calculated:

(10) $$V_{r}=\frac{0.87+1.12u}{1+u}\sqrt{\frac{E}{\rho }\left(\frac{1}{2\left(1+u\right)}\right)}.$$

where *u* is the Poisson’s ratio of the material, *ρ* is the material density, and *E* is the material’s elastic modulus.

The surface wave propagation delay $t_{1}$ from the exciting sensor position (*x_i_*, *z_i_*) to the receiving sensor position (*x_j_*, *z_j_*) can be expressed as

(11) $$t_{1}=\sqrt{\frac{{\left(x_{i}-x_{j}\right)}^{2}+{\left(z_{i}-z_{j}\right)}^{2}}{V_{r}}}.$$

The surface signal of the experimental signal can be removed by windowing the experimental signal using Equation (12).

(12) $$S_{ij}^{\prime }=\{\begin{array}{c} \varepsilon S_{ij}\left(t\right),\;\;\;\;\;\;\;\;\;\;t_{1}\leq t\leq t_{2}\\ S_{ij}\left(t\right),\;\;\;\;\;\;\;\;\;\;t<t_{1},t>t_{2} \end{array}.$$

In the equation, $S_{ij}^{\prime }$ is the signal after removing the surface wave, *S_ij_* is the original signal, and *ε* is the suppression coefficient. $t_{1}$ is the surface wave delay and $t_{2}$ is the time domain width of the excitation signal.

**Figure 14:**
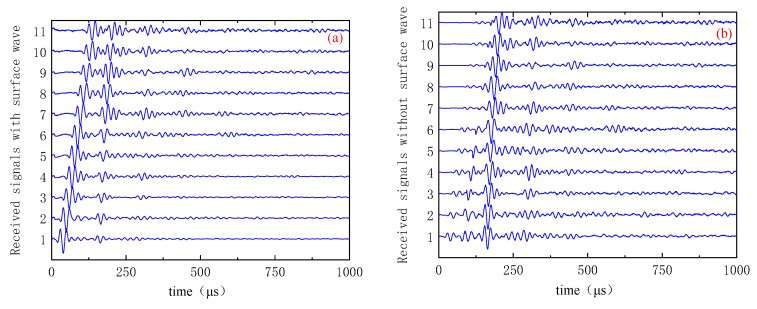
Time-domain images: (**a**) original signal and (**b**) signal after surface wave removal.

Figure 13b shows the time domain of the signal after the surface wave signal is removed by channel 1 and the signal from channel 4 is received. It can be seen that this method better suppresses the influence of surface wave signals. Figure 14 depicts the time domain of the signal received by channel 1 as the excitation source, the signal received by channels 2–12, and the signal after the surface wave signal is removed. It can be seen that the surface wave signals in the 11 groups of signals are well suppressed.

### 4.3. Analysis of Experimental Results

**Figure 15:**
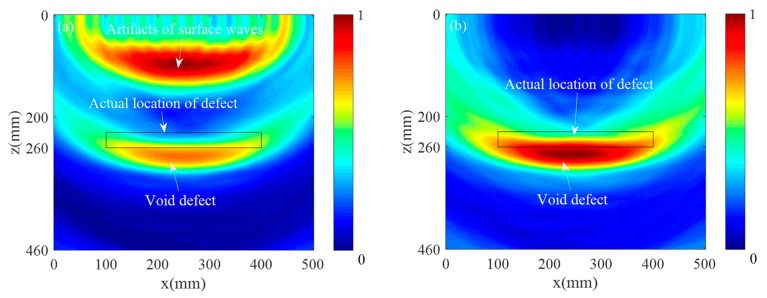
SAFT imaging results: (**a**) before surface wave suppression and (**b**) after surface wave suppression.

**Figure 16:**
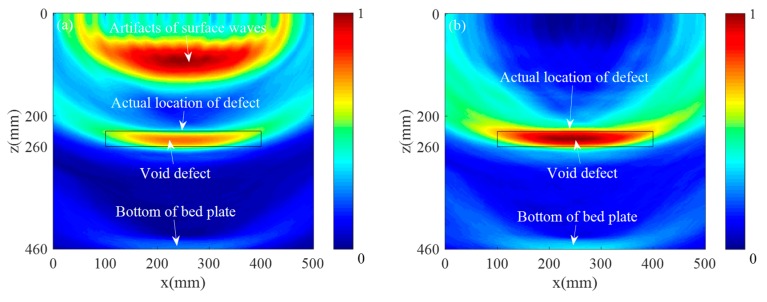
Multilayer SAFT imaging results: (**a**) before surface wave suppression and (**b**) after surface wave suppression.

**Figure 17:**
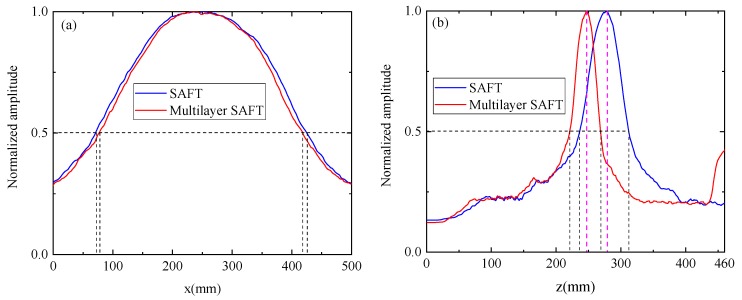
Sectional view of the imaging results: (**a**) *x*-direction and (**b**) *z*-direction.

The traditional SAFT imaging algorithm and the Multilayer SAFT imaging algorithm were used to perform ultrasonic imaging results on 66 sets of signal data collected by the MIRA-A1040 concrete ultrasonic tomography scanner in the pre-embedded void defect area. Figure 15 shows the imaging result obtained using the conventional SAFT imaging algorithm. Figure 15a shows the imaging result using the acquired original signal, and Figure 15b shows the imaging result using the signal after the removal of the surface wave. Figure 16 depicts the imaging result obtained by the Multilayer SAFT imaging algorithm. Specifically, Figure 16a shows the imaging result using the acquired original signal, and Figure 16b shows the imaging result using the signal after the removal of the surface wave. It can be seen from the imaging results of Figure 15b and Figure 16b that the Multilayer SAFT imaging algorithm fully considers the propagation speed of the ultrasonic wave in the track slab, mortar layer, and bedplate, making it more accurate than the traditional SAFT algorithm in terms of locating the void defect in the ballastless track structure. In addition, the presence of surface waves produces large artifacts in the imaging results, causing the imaging values of the depletion defects to be relatively low. After the surface waves are suppressed, the imaging quality of the depletion defects is obviously improved. A cross-sectional view of the imaging results of the two methods in the *x* and *z*-directions is shown in Figure 17. As can be seen in Figure 17a, the defect length range in the imaging results using the conventional SAFT imaging algorithm is (71.6 mm, 424.9 mm) with an error of 53.3 mm, the center position of the length is *x* = 244.5 mm, and the error is 5.5 mm. Using the Multilayer SAFT imaging algorithm, the defect length range is (78.3mm, 417.2mm), the error is 38.9 mm, the length center position is *x* = 244.5 mm, and the error is 5.5 mm. It can be seen from Figure 17b that the height range of the imaging result using the conventional SAFT algorithm is (218.9 mm, 309.1 mm), the error is 46.2 mm, the center position in the *z*-direction is *z* = 278.8 mm, and the error is 33.8 mm. The height range of the imaging structure using the Multilayer SAFT imaging algorithm is (218 mm, 265.3 mm) with an error of 17.3 mm, the center position in the *z*-direction is *z* = 248.1 mm with an error of 3.1 mm. From the above results, it is clear that the Multilayer SAFT imaging algorithm, which considers the propagation wave velocity in different media, is significantly better than the SAFT imaging algorithm using single wave velocity. The length characterization accuracy is improved by 27% and the height characterization accuracy is improved by 62.6%. Furthermore, the positioning accuracy of the center position in the *z*-direction is increased by 90.8%.

## 5. Conclusions

With the aim of solving the high-precision ultrasonic imaging problem of detecting void defects in ballastless track structures, this paper combines ray tracing technology and the SAFT imaging algorithm to propose a Multilayer SAFT imaging algorithm. The finite element simulation and experimental results show that the Multilayer SAFT imaging algorithm can improve imaging precision of void defects. The specific results are as follows:

(1) Based on the shortest path principle of Fermat, the function of the position of the refraction point and the propagation path of the forward tracking ultrasonic wave were obtained. Thereby, the tracking of the propagation path of the acoustic wave in the ballastless track structure was realized, and the propagation delay of the ultrasonic wave in the ballastless track structure could be accurately calculated.
(2) A two-dimensional finite element model of ultrasonic wave propagation in a ballastless track structure was established. The ultrasonic signal matrix was captured in the finite element model. The acquired signal was ultrasonically imaged by conventional SAFT imaging and the multilayer SAFT imaging method, separately. Compared with traditional SAFT imaging, this method improved the length characterization accuracy by 32.9%, the height characterization accuracy by 93.2%, and the positioning accuracy in the *z*-direction center position by 93.2%.
(3) A model of a ballastless track structure with a 1:1 ratio was constructed in the laboratory. Ultrasonic echo signals were acquired by a MIRA-A1040 concrete ultrasonic tomography scanner. The data were exported and processed in MATLAB software. The experimental results showed that, according to the characteristics of the time domain of the experimental signal, selecting a reasonable window function can effectively remove the influence of surface waves. The Multilayer SAFT imaging method provided better accuracy determination in the two directions (the length and height) of the void defect. These results were significantly improved compared with those achieved by the traditional SAFT algorithm. The length characterization accuracy was improved by 27%, the height characterization accuracy was improved by 62.6%, and the positioning accuracy in the *z*-direction center position was improved by 90.8%.

In summary, the Multilayer SAFT algorithm fully considers the different propagation speeds of the ultrasonic wave in different layers of a ballastless track structure and thus improves imaging precision of void defects. In this way, it can overcome the problem of the traditional of SAFT imaging algorithm, which is not applicable for the imaging of void defects in multilayer ballastless track structures. 

## Figures and Tables

**Table 1:** Material parameters of each layer in the ballastless track structure.

Property	Track Slab	Mortar Layer	Bed Plate	Void Area
Density (kg/m^3^)	2500	1800	2500	1.29 (air density)
Width (mm)	500	500	500	300
Thick (mm)	200	60	200	30
Shear wave velocity (m/s)	2466	1521	2466	0

**Table 2:** Finite element model parameters.

Model Size	500 × 460 mm
Transducer spacing	30 mm
Exciting frequency	50 kHz
Grid size	0.425 × 0.425 mm
Sampling frequency	10 MHz

## References

[1] Tang J.L. (2019). China Railway (CR): Chinese High-Speed Rail Operating Mileage Reached More Than 29,000 Kilometers [EB/OL]. http://news.china.com.cn/txt/2019-01/02/content_74333468.htm.

[2] Zhao G.T. (2019). Research and application of general constructions technologies for high-speed railway in China. J. China Railw. Soc..

[3] Fan G.P., Zhang H.Y., Zhu W.F., Zhang H., Chai X.D. (2019). Numerical and experimental research on identifying a delamination in ballastless slab track. Materials.

[4] Xu J.M., Wang P., An B.Y. (2018). Damage detection of ballastless railway tracks by the impact-echo method. Proc. Inst. Civ. Eng. Transp..

[5] Cosmes-Lopez M.F., Castellanos F., Cano-Barrita P.F.D. (2017). Ultrasound frequency analysis for identification of aggregates and cement paste in concrete. Ultrasonics.

[6] Xu B., Chen H.B., Song X. (2017). Numerical study on the mechanism of active interfacial debonding detection for rectangular CFSTs based on wavelet packet analysis with piezoceramics. Mech. Syst. Signal Process..

[7] Luo M.Z., Li W.J., Hei C. (2016). Concrete infill monitoring in concrete-filled FRP tubes using a PZT-based ultrasonic time-of-flight method. Sensors.

[8] Xu B., Li B., Song G.B. (2013). Active debonding detection for large rectangular CFSTs based on wavelet packet energy spectrum with piezoceramics. J. Struct. Eng..

[9] Zhu W., Xiang Y., Liu C.J., Deng M., Xuan F.Z. (2018). A feasibility study on fatigue damage evaluation using nonlinear Lamb waves with group-velocity mismatching. Ultrasonics.

[10] Zhu W.F., Zhang H.Y., Liu F.J. (2019). Lamb waves topological imaging of multiple blind defects in an isotropic plate. Int. J. Acoust. Vib..

[11] Liu H., Xia H.Y., Zhuang M.W. (2019). Reverse time migration of acoustic waves for imaging based defects detection for concrete and CFST structures. Mech. Syst. Signal Process..

[12] Freeseman K., Khazanovich L., Hoegh K. (2016). Nondestructive monitoring of subsurface damage progression in concrete columns damaged by earthquake loading. Eng. Struct..

[13] Khazanovich L., Hoegh K. (2016). Quantitative ultrasonic evaluation of concrete structures using one-sided access. American Institute of Physics Conference Series.

[14] White J., Hurlebaus S., Shokouhi P., Wimsatt A. (2014). Use of ultrasonic tomography to detect structural impairment in tunnel linings. Transp. Res. Rec..

[15] Sherwin C.W., Ruina J.P., Rawcliffe R.D. (1962). Some early developments in synthetic aperture radar systems. Mil. Electron. IRE Trans..

[16] Frederick J.R., Vanden Broek C., Ganapathy S., Elzinga M.B., de Vries W., Papworth D., Hamano N. (1979). Improved Ultrasonic Nondestructive Testing of Pressure Vessels.

[17] Sternini S., Liang A.Y., Di S. (2019). Rail defect imaging by improved ultrasonic synthetic aperture focus techniques. Mater. Eval..

[18] Wu H.T., Chen J., Yang K.J. (2016). Ultrasonic array imaging of multilayer structures using full matrix capture and extended phase shift migration. Meas. Sci. Technol..

[19] Lin S.B., Shams S., Choi H.J. (2018). Ultrasonic imaging of multi-layer concrete structures. NDT E Int..

[20] He Y.L., Shen J.K., Li Z.W. (2019). Fractal characteristics of transverse crack propagation on CRTSII type track slab. Math. Probl. Eng..

[21] Wu S.W., Skjelvareid M.H., Yang K., Chen J. (2015). Synthetic aperture imaging for multilayer cylindrical object using an exterior rotating transducer. Rev. Sci. Instrum..

[22] Zheng S.B., Zhong Q.W., Chai X.D. (2018). A novel prediction model for car body vibration acceleration based on correlation analysis and neural networks. J. Adv. Transp..

[23] Zhu W.J., Deng M.X., Xiang Y.X., Xuan F.Z., Liu C.J., Wang Y.N. (2016). Modeling of ultrasonic nonlinearities for dislocation evolution in plastically deformed materials: Simulation and experimental validation. Ultrasonics.

[24] Skjelvareid M.H., Birkelund Y., Larsen Y. (2013). Internal pipeline inspection using virtual source synthetic aperture ultrasound imaging. NDT E Int..

[25] Fredrik L., Tomas O., Tadeusz S. (2003). Synthetic aperture imaging using sources with finite aperture: Deconvolution of the spatial impulse response. J. Acoust. Soc. Am..

[26] Skjelvareid M.H., Olofsson T., Birkelund Y. (2011). Synthetic aperture focusing of ultrasonic data from multilayered media using an omega-k algorithm. IEEE Trans. Ultrason. Ferroelectr. Freq. Control.

[27] Johnson J.A., Barna B.A. (1983). The effects of surface mapping corrections with synthetic-aperture focusing techniques on ultrasonic-imaging. IEEE Trans. Sonics Ultrason..

[28] Zhang H., Zhang H.Y., Zhang J.Y., Liu J.Q., Zhu W.F., Fan G.P., Zhu Q. (2019). Wavenumber imaging of near-surface defects in rails using green’s function reconstruction of ultrasonic diffuse fields. Sensors.

